# HKDC1 C-terminal based peptides inhibit extranodal natural killer/T-cell lymphoma by modulation of mitochondrial function and EBV suppression

**DOI:** 10.1038/s41375-020-0801-5

**Published:** 2020-03-23

**Authors:** Qi Chen, Jia Feng, Jinhu Wu, Zhendong Yu, Wei Zhang, Yonggang Chen, Paul Yao, Hongyu Zhang

**Affiliations:** 1 Department of Hematology, Peking University Shenzhen Hospital, Shenzhen, 518036 PR China; 2 Department of Pharmacology, Tongren Hospital of Wuhan University, Wuhan, 430060 PR China; 3 Central Laboratory, Peking University Shenzhen Hospital, Shenzhen, 518036 PR China; 4 Shenzhen Peking University-The Hong Kong University of Science and Technology Medical Center, Shenzhen, 518036 PR China

**Keywords:** Cancer metabolism, Cell signalling

## Abstract

Extranodal nasal-type natural killer/T-cell lymphoma (ENKTL) is an Epstein–Barr virus (EBV) associated lymphoma that progresses rapidly and relapses frequently. Advanced ENKTL is multidrug chemoresistant and has a poor prognosis. In this study, we aim to develop a novel hexokinase domain component 1 (HKDC1)-based antitumor target for ENKTL that is involved with the antimetabolic signaling pathway, EBV replication, and P-glycoprotein (P-gp) expression. We showed that HKDC1 is highly upregulated in ENKTL cells and HKDC1 knockdown significantly suppresses ENKTL tumor growth. In addition, HKDC1 is highly identical with four other hexokinase isoforms, with the only difference being in the last eight amino acids (aa) at the C-terminal. Further investigation showed that peptide delivery of the last eight aa of HKDC1 at the C-terminal (HKC8) with D-configuration using transferrin (Tf) receptor internalization sequence (Tf-D-HKC8) inhibits HKDC1 association with vascular endothelial growth factor 1 (VDAC1), resulting in mitochondrial dysfunction and reactive oxygen species (ROS) overgeneration and subsequently suppressing EBV replication and P-gp expression, making it very effective in killing EBV-positive ENKTL cells. Further in vivo experiments showed that local injection of Tf-D-HKC8 peptide significantly suppresses ENKTL tumor growth and EBV replication in ENKTL xenograft mouse models. We conclude that HKDC1 C-terminal-based peptides inhibit ENKTL by modulation of mitochondrial function and EBV suppression.

## Introduction

Extranodal natural killer/T-cell lymphoma (ENKTL) is an aggressive non-Hodgkin lymphoma commonly present in the nasal cavity [1–3]. It is most prevalent in Asian populations and is strongly associated with Epstein–Barr (EBV) infection [4, 5]. Advanced ENKTL progresses rapidly and relapses inevitably with multidrug chemoresistance, resulting in poor survival rates in just a few months. Currently, development of targeted therapy for ENKTL is still urgently needed [6, 7].

ENKTL is involved in multiple pathogenic pathways, such as JAK/STAT, PDGF, Aurora kinase, NFκB, and the c-Myc pathway, making it difficult to identify potential molecular targeting therapies for ENKTL [8]. ENKTL is characterized by EBV latent infection with the expression of EBNA1 and LMP1, and specific inhibitors for these viral proteins have been found to suppress EBV-dependent tumor growth in xenograft models. In addition, EBV infection directly or indirectly upregulates the expression of P-glycoprotein (P-gp) [9–11] and programmed death ligand 1 [6, 12]; targeting of either of these molecules has significantly improved the poor prognosis of ENKTL. However, ENKTL still relapses eventually [13], indicating that these methods cannot interrupt the potential original driving force that was triggered by EBV DNA. This makes EBV DNA a novel potential therapeutic target in ENKTL treatment [8, 14].

Hexokinase (HK) is a rate-limiting enzyme that catalyzes the phosphorylation of hexose sugars and subsequently regulates glucose metabolism [15]. Four kinds of HK isoforms, including HK1, HK2, HK3, and HK4, have been previously well characterized [16, 17]. Recently, a new HK isoform named HK domain component 1 (HKDC1) has been found to be involved with glucose homeostasis [18, 19] and tumor development [20–23].

In this study, we aim to develop a novel HKDC1-based antitumor target for ENKTL that is involved with the antimetabolic signaling pathway, EBV replication, and P-gp expression. Our preliminary data showed that HKDC1 expression is highly upregulated in ENKTL cells and HKDC1 knockdown significantly suppresses ENKTL tumor growth, indicating that HKDC1 plays an important role in ENKTL tumor development. The amino acid (aa) alignment shows that HKDC1 has a sequence that is highly identical with four other HK isoforms, with the only difference being in the last eight aa at the C-terminal [24]. Overexpression of the last eight aa of HKDC1 at the C-terminal (HKC8) truncates significantly suppresses tumor growth and results in dissociation of HKDC1 from vascular endothelial growth factor 1 (VDAC1), indicating that HKC8 may be a novel antitumor target for ENKTL treatment. Further investigation showed that delivery of the HKC8 peptide to ENKTL cells using transferrin (Tf) receptor internalization sequence (Tf-D-HKC8) induces apoptosis and oxidative stress with subsequent DNA damage [25, 26]. Further in vivo experiments showed that local injection of Tf-D-HKC8 peptide significantly suppresses EBV replication and P-gp expression, making it very effective in suppressing EBV-positive ENKTL tumor growth in xenograft mouse models. We conclude that peptide HKC8 dissociates HKDC1 from VDAC1, interrupts glucose metabolism, and induces reactive oxygen species (ROS) overgeneration, resulting in ENKTL cell apoptosis, EBV DNA damage, and P-gp suppression. Thus, HKC8 is a novel therapeutic target for ENKTL antitumor drug development.

## Materials and methods

An expanded “Materials and Methods” section is available in Supplemental Information Data S1, and primers used in this study were shown in Table S1.

### HK activity assay

Total HK activity from cell lysates was measured as the glucose phosphorylating capacity of whole cell extracts using a standard G6PDH-coupled assay [27, 28]. The glucose and ATP-dependent reduction of NADP was monitored by a 96-well microplate reader at 340 nm in the presence of excess G6PDH. All assays (final assay mixture composition: 1 U/ml G6PDH, 0.5 mg/ml NADP, 6.7 mM ATP, 7.7 mM MgCl2, 4.0 mM glucose, 45 mM KCl, 1 mM NaH2PO4, 10.6 mM monothioglycerol, 0.01% Triton X-100, 0.5 mM EDTA, and 42 mM Tris HCl, pH 8.5) were performed at 25 °C under conditions of linear HK-limited NADPH formation. Total HK activity was normalized for cellular protein content and was expressed in enzyme activity units corresponding to the glucose phosphorylation rate in micromoles per minute [29].

### In vivo mouse protocol

To measure malignant SNK6 xenograft growth, 100 µl of 1 × 10^5^ SNK6 cells in PBS were mixed with 100 µl growth factor-reduced basement membrane matrix (Matrigel™, BD Biosciences) resulting in 200 µl of solution that was injected subcutaneously along the mouse flank at each intended tumor site. Tumor size was measured using a digital caliper and volume was calculated, and mice were randomly divided into four groups (*n* = 9 per group) when the tumor volume reached 50–100 mm^3^ on day ~18 after tumor inoculation and given treatment as follows: Group 1 (control (CTL)) was intratumorally injected with HBSS buffer (5.33 mM KCl, 0.44 mM KH2PO4, 138 mM NaCl, 4 mM NaHCO3, 0.3 mM Na2HPO4, and 5.6 mM glucose, pH 7.3) containing 0.05% DMSO; Group 2 (Tf-D-HKN15) received HBSS buffer containing 0.05% DMSO with 50 µM Tf-D-HKN15 peptides; Group 3 (Tf-D-HKC15) received HBSS buffer containing 0.05% DMSO with 50 µM Tf-D-HKC15 peptides; Group 4 (Tf-D-HKC8) received HBSS buffer containing 0.05% DMSO with 50 µM Tf-D-HKC8 peptides. The xenografts were injected (two points, 20 µl per tumor) every 2 days. Beginning on the day of inoculation, mouse weight and tumor volume were monitored every 2 days. Mice were monitored for changes in body weight and sacrificed when values fell below 20% of their initial weight; the survival curve was calculated and the final tumor tissues were isolated for biomedical analysis.

### Biomedical analysis of tumor tissues

Parts of the tumor tissues were fixed in 4% buffered formaldehyde, paraffin embedded, and sectioned to 4 mm thickness. They were then either processed for immunohistochemistry (IHC) or histopathological analyses were performed with H&E staining. Images were taken using a Carl Zeiss MIRAX MIDI slide scanner, and analyses were performed using a 3DHISTECH Pannoramic Viewer. Part of the tumor tissues were isolated for in vivo monitoring of superoxide anion release, gene expression was measured through real time PCR for mRNA and western blotting for protein levels, the copies of the EBV genome were measured by real time PCR, and the binding of HKDC1 and VDAC1 was evaluated by immunoprecipitation (IP)/WB [30].

## Results

### HKDC1 expression regulates tumor growth in ENKTL cells

**Fig. 1:**
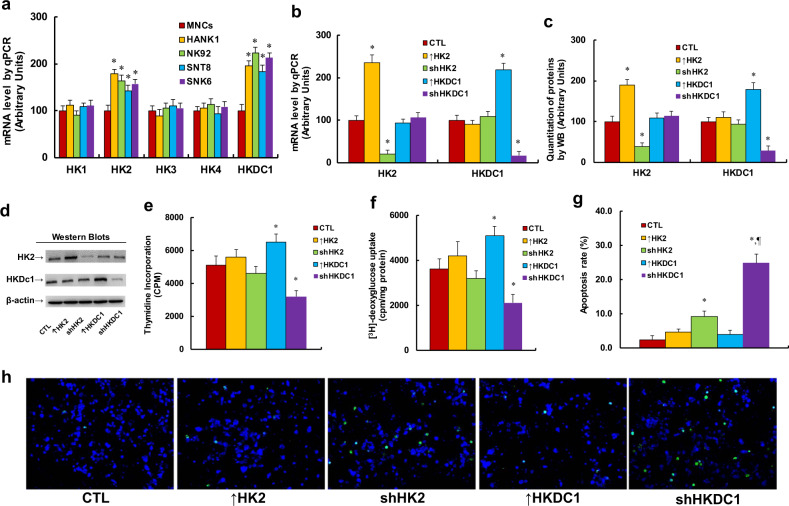
HKDC1 expression regulates tumor growth in ENKTL cells. **a** Different ENKTL cell lines were used for gene expression analysis of hexokinase, *n* = 4. *P < 0.05, vs MNCs group. **b**–**g** The SNK6 cells were infected by empty lentivirus control (CTL), HK2 (↑HK2) or HKDC1 (↑HKDC1) overexpression lentivirus, or HK2 (shHK2) or HKDC1 (shHKDC1) knockdown lentivirus, and the cells were used for biomedical analysis. **b** mRNA expression by qPCR, *n* = 4. **c** Protein quantitation by western blotting, *n* = 5. **d** Representative pictures for **c**. **e** Cell proliferation analysis by thymidine incorporation, *n* = 5. **f** [^3^H]-deoxyglucose uptake, *n* = 5. **g** Apoptosis analysis by TUNEL assay, *n* = 4. **h** Representative picture for **g**. *P < 0.05, vs CTL group; ^¶^*P* < 0.05, vs shHK2 group. Data were expressed as mean ± SEM.

We first measured the mRNA expression levels of five different HKs (including HK1, HK2, HK3, HK4, and HKDC1) in different ENKTL cell lines (including HANK1, NK92, SNT8, and SNK6) compared with healthy MNCs (see Fig. 1a). The results showed that the expression of HK1, HK3, and HK4 did not change, while HK2 mRNA levels increased to 179%, 164%, 143%, and 157%, respectively; in addition, HKDC1 mRNA levels increased to 196%, 223%, 184%, and 213%, respectively, in HANK1, NK92, SNT8, and SNK6 cells compared with MNCs. The results indicate that the expression of HK2 and/or HKDC1 may contribute to tumor growth in ENKTL cells. We then investigated the potential role of HK2 and HKDC1 in SNK6 cells by either overexpression or knockdown of HK2/HKDC1. The findings showed that HK2 overexpression (↑HK2) increased and HK2 knockdown (shHK2) decreased HK2 mRNA levels to 236% and 21%, respectively, while HKDC1 overexpression (↑HKDC1) increased and HKDC1 knockdown (shHKDC1) decreased HKDC1 mRNA to 219% and 17%, respectively, compared with CTL cells (see Fig. 1b). We then measured the protein levels for these genes, and an expression pattern similar to that of the mRNA levels was observed (see Fig. 1c, d). The results indicate that manipulation of the expression of HK2 and HKDC1 using lentivirus is successful and efficient. We then evaluated the effect of HK2/HKDC1 expression on tumor growth through thymidine incorporation (see Fig. 1e) and [^3^H]-deoxyglucose uptake (see Fig. 1f). The findings showed that HK2 expression had little effect; HKDC1 overexpression (↑HKDC1) increased both thymidine incorporation and [^3^H]-deoxyglucose uptake to 127% and 141%, respectively, while HKDC1 knockdown (shHKDC1) decreased both thymidine incorporation and [^3^H]-deoxyglucose uptake to 63% and 58%, respectively, compared with the CTL group. Finally, we evaluated the effect of HK2/HKDC1 expression on apoptosis using TUNEL assay (see Fig. 1g, h). The results showed that overexpression of both HK2 (↑HK2) and HKDC1 (↑HKDC1) had little effect, while knockdown of HK2 (shHK2) and HKDC1 (shHKDC1) increased apoptosis rates by 3.83- and 10.4-fold, respectively, compared with the CTL group. Our results indicate that HKDC1 expression plays a dominant role in regulation of tumor growth in ENKTL cells, while the effect of HK2 is very small.

### Overexpression of the last eight aa of HKDC1 at the C-terminal truncate (↑HKDC1-Δc8a) suppresses tumor growth in ENKTL cells

**Fig. 2:**
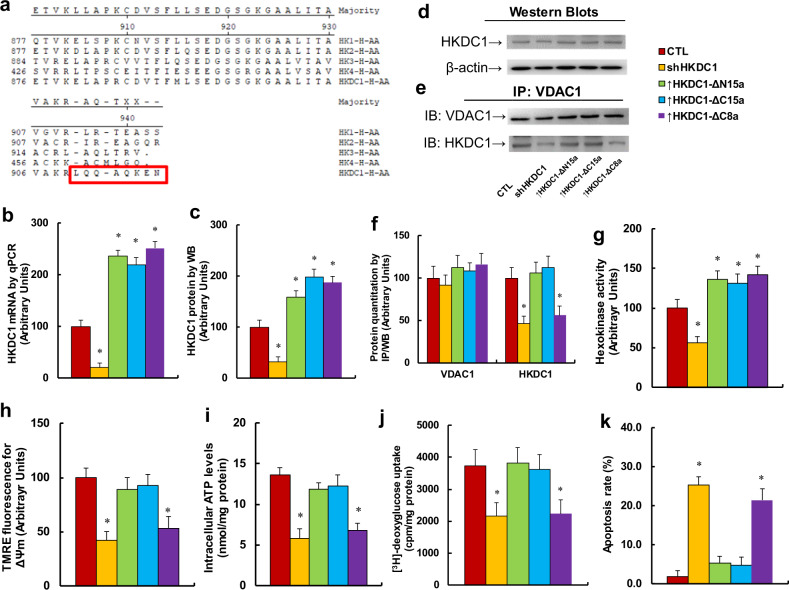
Overexpression of the last eight amino acids of HKDC1 at the C-terminal truncate (↑HKDC1-Δc8a) suppresses tumor growth in ENKTL cells. **a** The amino acid (aa) sequence alignment for five human hexokinase isoforms. **b**–**k** The SNK6 cells were either infected by empty lentivirus control (CTL), HKDC1 knockdown lentivirus (shHKDC1), or expression lentivirus for the last 15 aa of HKDC1 at the N-terminal truncate (↑HKDC1-ΔN15a) the last 15 aa of HKDC1 at the C-terminal truncate (↑HKDC1-ΔCN15a), or the last eight aa of HKDC1 at the C-terminal truncate (↑HKDC1-ΔC8a). The cells were used for biomedical analysis. **b** mRNA analysis for HKDC1, *n* = 4. **c** Quantitation of HKDC1 protein by western blots, *n* = 5. **d** Representative picture for **c**. **e** Representative picture for IP/WB analysis. **f** Quantitation of IP/WB analysis for HKDC1/VDAC1 binding, *n* = 5. **g** Hexokinase activity assay, *n* = 5. **h** Mitochondrial membrane potential (ΔΨm), *n* = 5. **i** Intracellular ATP levels, *n* = 5. **j** [^3^H]-deoxyglucose uptake, *n* = 5. **k** Apoptosis analysis by TUNEL assay, *n* = 4. *P < 0.05, vs CTL group. Data were expressed as mean ± SEM.

We investigated the potential role of HKDC1 in ENKTL tumor growth. The aa sequence of HKDC1 was aligned with other four HK isoforms (see Fig. 2a). Our findings showed that the HKDC1 sequence is highly identical with that of the other isoforms, with the only difference being in the last eight aa in the C-terminal, indicating that the last eight aa may play an important role in tumor growth. We then truncated several terminal aa sequences of HKDC1, including the last 15 aa at the N-terminal (HKDC1-ΔN15a), the last 15 aa at the C-terminal (HKDC1-ΔC15a), and the last eight aa at the C-terminal (HKDC1-ΔC8a), packaged them using lentivirus vector, and infected the SNK6 cells to see the potential effects. As shown in Fig. 2b, the results indicated that shHKDC1 decreased HKDC1 mRNA levels to 21%, while infection of HKDC1-ΔN15a, HKDC1-ΔC15a, and HKDC1-ΔC8a increased HKDC1 mRNA to 236%, 219%, and 251%, respectively, compared with the CTL group. We also measured the protein levels in these cells, and an expression pattern similar to that of the mRNA levels was observed (see Fig. 2c, d). This indicates that the manipulation of HKDC1 expression by lentivirus was successful and efficient. We then measured the binding of HKDC1 with VDAC1 using IP techniques. The results showed that there was little effect on the HKDC1-ΔN15a and HKDC1-ΔC15a groups, while binding of HKDC1 with VDAC1 in the shHKDC1 and HKDC1-ΔC8a groups decreased to 46% and 56%, respectively, (see Fig. 2e, f). We also measured the total HK activity (see Fig. 2g). The findings showed that HK activity in the shHKDC1 group decreased to 54%, while infection of HKDC1-ΔN15a, HKDC1-ΔC15a, and HKDC1-ΔC8a increased HKDC1 activity to 136%, 131%, and 142%, respectively. Our results indicate that truncate of the last eight aa at the HKDC1 C-terminal does not significantly affect HK activity; instead, it suppresses the binding of HKDC1 with VDAC1. We also measured the effect of HKDC1-ΔC8a on mitochondrial function, including mitochondria membrane potential (ΔΨm) and intracellular ATP generation. Our findings showed that shHKDC1 and HKDC1-ΔC8a decreased ΔΨm to 42% and 53%, respectively, (see Fig. 2h); in addition, it decreased ATP generation to 43% and 50%, respectively, (see Fig. 2i), while treatment of HKDC1-ΔN15a and HKDC1-ΔC15a had no effect. We then measured [^3^H]-deoxyglucose uptake (see Fig. 2j), and the results showed that shHKDC1 and HKDC1-ΔC8a decreased uptake to 66% and 68%, respectively, while treatment of HKDC1-ΔN15a and HKDC1-ΔC15a had no effect. We finally measured the apoptosis rate (see Fig. 2k). The results showed that the apoptosis rate in shHKDC1 and HKDC1-ΔC8a increased by 13.4- and 11.3-fold, respectively, while treatment of HKDC1-ΔN15a and HKDC1-ΔC15a had no effect. This observation that HKDC1-∆C15a is not as effective as HKDC1-∆C8a may be because truncation of last 15 aa of HKDC1 (HKDC1-∆C15a) significantly changed the function and structure of HKDC1, subsequently causing it to be unable to compete with endogenous HKDC1 to bind with VDAC1. Our results suggest that the last eight aa of HKDC1 at the C-terminal play an important role in regulation of HKDC1 binding with VDAC1 in ENKTL cells.

### Delivery of the peptide for the last eight aa of HKDC1 at the C-terminal (Tf-D-HKC8) induces cell death in EBV-positive ENKTL cells

We investigated the potential role of HKDC1-based peptides in ENKTL cells that were isolated from the MNCs of ENKTL patients or SNK6 cell line. Three different kinds of HKDC1-based targeting peptides, including the last 15 aa at either the N-terminal (HKN15) or C-terminal (HKC15), or the last eight aa at the C-terminal (HKC8) were selected; four different kinds of cell-penetrating sequences were employed to deliver the HKDC1-based peptides, including Antp (a 16 penetrating residue long sequence from the *Drosophila* antennapedia-homeodomain), penetration-accelerating segment, TAT (HIV-1 TAT sequence), and Tf (the Tf receptor (TfR) internalization sequence) sequences. All of the aa with the D-configuration (marked as D) were synthesized as retro-inverso peptides. These peptides were used to treat either ENKTL cells that were isolated from the MNCs of ENKTL patients or SNK6 cells using different concentrations of peptides (0.1–15 µM) for 6 h, and then the half-maximal cell death activity (IC_50_) value was calculated (see details in Table 1). Our results showed that peptide Tf-D-HKC8 seems to be the most efficient in killing ENKTL cells, and the cell-penetrating sequence Tf is the most effective delivery agent. We then evaluated the toxic effect of two peptides (including Tf-D-HKC15 and Tf-D-HKC8) on different kinds of primary and cancer cell lines (see details in Table 2). Our results showed that peptide Tf-D-HKC8 was much more effective in killing cells than peptide Tf-D-HKC15, indicating that HKDC1-based peptide for the last eight aa (but not the last 15 aa) at the C-terminal plays a very important role in suppressing the cells. In addition, both peptides have little cytotoxicity to either primary HMECs or healthy MNCs, indicating that the peptides have no obvious off-target effect. We also evaluated the peptide delivery efficiency using ^125^I-labeled uptake assay for both Tf-D-HKC8 and Tf-D-HKC15 peptides. The results showed that 53.4% of peptide Tf-D-HKC8 was delivered into SNK6 cells after 6-h incubation, while only 35.6% of peptide Tf-D-HKC15 was delivered, which may be due to the significant difference in molecular weight of the two peptides (see Fig S1). Furthermore, the suspension cells were more susceptible to peptide Tf-D-HKC8-induced cytotoxicity than adherent cells. Very interestingly, the EBV-positive suspension cells seems more susceptible than EBV-negative suspension cells (see Table 2). Our results suggest that peptide Tf-D-HKC8 induces significant cell death in ENKTL cells, especially in EBV-positive suspension cells.

**Table 1:** HKDC1-based peptides induce cell death in MNCs from ENKTL patients and SNK6 cells.

Peptide name	Peptide sequence	AA	IC_50_ (µM)	
			ENKTL	SNK6
Antp-HKC8	RQIKIWFQNRRMKWKK-*LQQAQKEN*	24	1.3 ± 0.4 (*n* = 3)	1.3 ± 0.3 (*n* = 4)
Antp-D-HKC8	**RQIKIWFQNRRMKWKK**-*LQQAQKEN*	24	1.0 ± 0.3 (*n* = 3)	1.2 ± 0.4 (*n* = 3)
PAS-HKC8	GKPILFF-*LQQAQKEN*	15	1.4 ± 0.4 (*n* = 4)	1.4 ± 0.3 (*n* = 3)
PAS-D-HKC8	**GKPILFF**-*LQQAQKEN*	15	1.1 ± 0.3 (*n* = 3)	1.3 ± 0.3 (*n* = 4)
TAT-HKC8	GYGRKKRRQRRRG-*LQQAQKEN*	21	1.9 ± 0.4 (*n* = 4)	2.0 ± 0.4 (*n* = 3)
TAT-D-HKC8	**GYGRKKRRQRRRG**-*LQQAQKEN*	21	1.5 ± 0.7 (*n* = 4)	1.7 ± 0.5 (*n* = 4)
Tf-HKC8	HAIYPRH-*LQQAQKEN*	15	0.5 ± 0.2 (*n* = 3)	0.7 ± 0.2 (*n* = 3)
Tf-Scram	HAIYPRH-*EILNKEKK*	15	>15.0 (*n* = 3)	>15.0 (*n* = 3)
Tf-D-HKC8	**HAIYPRH**-*LQQAQKEN*	15	0.2 ± 0.1 (*n* = 5)	0.3 ± 0.2 (*n* = 5)
Tf-D-Scram	**HAIYPRH**-*EILNKEKK*	15	>15.0 (*n* = 3)	>15.0 (*n* = 3)
Tf-HKC15	HAIYPRH-*ITAVAKRLQQAQKEN*	22	1.2 ± 0.4 (*n* = 3)	1.4 ± 0.3 (*n* = 3)
Tf-D-HKC15	**HAIYPRH**-*ITAVAKRLQQAQKEN*	22	0.9 ± 0.3 (*n* = 3)	1.2 ± 0.5 (*n* = 3)
Tf-D-Scram	**HAIYPRH**-*NAAAKLAVEILNKEK*	22	>15.0 (*n* = 3)	>15.0 (*n* = 3)
Tf-HKN15	HAIYPRH-*MFAVHLMAFYFSKLK*	22	>15.0 (*n* = 3)	>15.0 (*n* = 3)
Tf-D-HKN15	**HAIYPRH**-*MFAVHLMAFYFSKLK*	22	>15.0 (*n* = 3)	>15.0 (*n* = 3)

Cells were incubated with different concentrations of peptides (0.1–15 µM) for 6 h. The half-maximal cell death activity (IC50) value was determined by PI staining with subsequent FACS analysis. Results are expressed as mean ± SEM. The cell-penetrating sequence is underlined, the HKDC1-based targeting sequences are in italics, and the amino acids in the D-configuration are marked in bold.

*AA* amino acid numbers of peptides, *Antp* a 16 penetrating residue long sequence from the *Drosophila* antennapedia-homeodomain, *D* D-configuration, all the D-amino acids were synthesized as retro-inverso peptides, *HKC8* last eight aa of the C-terminal in human HKDC1, *HKC15* last 15 aa of the C-terminal in human HKDC1, *HKN15* first 15 aa of the N-terminal in human HKDC1, *IC50* half-maximal cell death activity value, *PAS* penetration-accelerating segment, *Scram* scramble peptide sequence, *TAT* HIV-1 TAT sequence, *Tf* transferrin receptor internalization sequence.

**Table 2:** HKDC1-based peptides induce cell death in different cancer cell lines.

Cell line	Cell type/EBV(+/−)	Peptide IC_50_ (µM)	
		Tf-D-HKC15	Tf-D-HKC8
**HMECs**	Human mammary epithelial cells/EBV(−)	>15.0 (*n* = 3)	>15.0 (*n* = 3)
**Healthy MNCs**	Healthy human mononuclear cells/EBV(−)	>15.0 (*n* = 3)	>15.0 (*n* = 3)
**SNK6**	Human natural killer/T-cell lymphoma/EBV(+)	1.2 ± 0.3 (*n* = 3)	0.3 ± 0.2 (*n* = 5)
**HANK1**	Human natural killer/T-cell lymphoma/EBV(+)	1.4 ± 0.6 (*n* = 3)	0.7 ± 0.4 (*n* = 3)
**SNT8**	Human natural killer/T-cell lymphoma/EBV(+)	1.1 ± 0.4 (*n* = 3)	0.6 ± 0.3 (*n* = 3)
**Namalwa**	Human Burkitt’s lymphoma/EBV(+)	0.9 ± 0.3 (*n* = 3)	0.7 ± 0.4 (*n* = 3)
**NK92**	Human natural killer non-Hodgkin’s lymphoma/EBV(−)	1.5 ± 0.6 (*n* = 3)	0.9 ± 0.4 (*n* = 3)
**MM.1R**	Human B lymphoblast/EBV(−)	2.3 ± 0.7 (*n* = 3)	1.6 ± 0.4 (*n* = 3)
**U266B1**	Human B lymphoblast/EBV(−)	2.2 ± 0.6 (*n* = 3)	1.4 ± 0.3 (*n* = 3)
**RPMI 8226**	Human B lymphoblast/EBV(−)	1.9 ± 0.7 (*n* = 3)	1.3 ± 0.6 (*n* = 3)
**Kasumi-1**	Human acute myeloblastic leukemia/EBV(−)	2.7 ± 0.9 (*n* = 3)	1.8 ± 0.6 (*n* = 3)
**HL-60**	Human acute promyelocytic leukemia/EBV(−)	2.2 ± 0.6 (*n* = 3)	1.7 ± 0.4 (*n* = 3)
**THP1**	Human monocytic leukemia/EBV(−)	2.3 ± 0.5 (*n* = 3)	1.8 ± 0.6 (*n* = 3)
*MCF7*	Human breast adenocarcinoma/EBV(−)	>15.0 (*n* = 3)	>15.0 (n = 3)
*MDA-MB-231*	Human breast adenocarcinoma/EBV(−)	14.4 ± 0.8 (*n* = 3)	11.6 ± 1.1 (*n* = 3)
*SW480*	Human colorectal adenocarcinoma/EBV(−)	8.9 ± 0.9 (*n* = 3)	6.7 ± 0.8 (*n* = 3)
*SW620*	Human colorectal adenocarcinoma/EBV(−)	12.5 ± 0.8 (*n* = 3)	10.2 ± 0.7 (*n* = 5)
*Hela*	Human cervix adenocarcinoma/EBV(−)	9.8 ± 0.6 (*n* = 3)	6.8 ± 0.9 (*n* = 3)
*HepG2*	Human hepatocellular carcinoma/EBV(−)	>15.0 (*n* = 3)	>15.0 (*n* = 3)

Cells were incubated with different concentrations of peptides (0.1–15 µM) for 6 h (for suspension cells, in bold), or 12 h (for adherent cells, in italics). Cell death IC50 was determined by PI staining with subsequent FACS analysis. Results are expressed as mean ± SEM.

### Peptide Tf-D-HKC8 inhibits EBV replication and P-gp expression through ROS generation and DNA damage in ENKTL cells

**Fig. 3:**
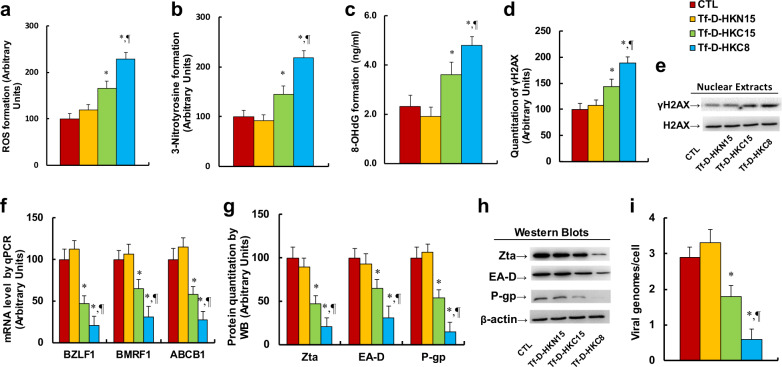
Peptide for the last eight aa of HKDC1 at the C-terminal (Tf-D-HKC8) inhibits EBV replication and P-gp expression through ROS generation and DNA damage in ENKTL cells. SNK6 cells were treated with control (CTL), Tf-D-HKN15, Tf-D-HKC15, or Tf-D-HKC8 peptide (0.5 µM) for 6 h and the cells were used for biomedical analysis. **a** ROS formation, *n* = 5. **b** 3-nitrotyrosine (3-NT) formation, *n* = 5. **c** 8-OHdG formation, *n* = 5. **d** Quantitation of γH2AX formation, *n* = 5. **e** Representative western blotting bands for **d**. **f** mRNA level by qPCR for BZLF1, BMRF1, and ABCB1, *n* = 4. **g** Protein quantitation for Zta, EA-D, and P-gp, *n* = 5. **h** Representative pictures of western blots for **g**. **i** EBV viral genomes/cell by qPCR, *n* = 4. *P < 0.05, vs CTL group; ^¶^*P* < 0.05, vs Tf-D-HKC15 group. Results were expressed as mean ± SEM.

We investigated the potential mechanism and cytotoxic effect of HKDC1-based peptides on EBV replication in SNK6 cells. We first evaluated peptide-mediated oxidative stress. The results showed that peptides Tf-D-HKC15 and Tf-D-HKC8 increased ROS formation to 165% and 229%, respectively, (see Fig. 3a), and increased 3-nitrotyrosine formation to 165% and 229%, respectively, (see Fig. 3b), compared with CTL group; peptide Tf-D-HKN15 had no effect. We also measured DNA damage. The results showed that peptides Tf-D-HKC15 and Tf-D-HKC8 increased 8-OHdG formation to 157% and 209%, respectively, (see Fig. 3c), and increased _γ_H2AX formation to 144% and 189%, respectively, (see Fig. 3d, e); again, peptide Tf-D-HKN15 showed no effect. We then measured the effect of peptides on EBV gene expression. The results showed that peptides Tf-D-HKC15 and Tf-D-HKC8 decreased BZLF1 mRNA to 47% and 21%, respectively; decreased BMRF1 mRNA to 65% and 31%, respectively; and decreased ABCB1 mRNA to 58% and 27%, respectively (see Fig. 3f). We also measured protein levels of Zta, EA-D, and P-gp (encoded by BZLF1, BMRF1, and ABCB1, respectively) in those cells, and an expression pattern similar to that of the mRNA levels was observed (see Fig. 3g, h). We finally measured the EBV genome copies. Our findings showed that peptides Tf-D-HKC15 and Tf-D-HKC8 decreased EBV genome copies to 62% and 21%, respectively, while peptide Tf-D-HKN15 showed no effect. In order to confirm that HKDC1-based peptide-mediated EBV suppression was due to oxidative stress and DNA damage, the SNK6 cells were infected by SOD2 expression lentivirus. The results showed that SOD2 overexpression (Tf-D-HKC8/SOD2) completely restored peptide Tf-D-HKC8-mediated increased ROS formation (see Fig. S2a) and 8-OHdG formation (Fig. S2b); in addition, it completely restored mRNA expression of BZLF1, BMRF1, and ABCB1 (see Fig. S2c) as well as peptide Tf-D-HKC8-induced EBV genome replication (see Fig. S2d). Our results indicate that peptide Tf-D-HKC8 inhibits EBV replication through ROS generation and DNA damage in ENKTL cells.

### Peptide Tf-D-HKC8 dissociates HKDC1 from VDAC1 and induces mitochondrial dysfunction and apoptosis in ENKTL cells

**Fig. 4:**
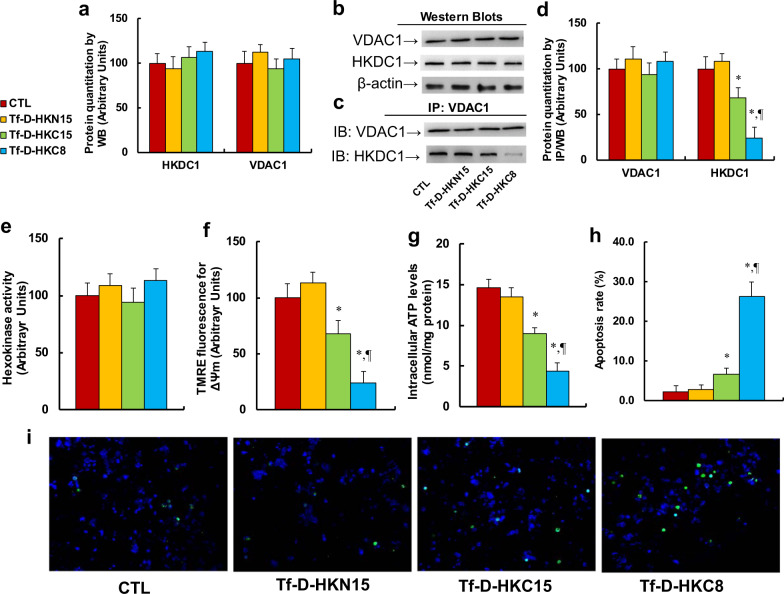
Peptide Tf-D-HKC8 dissociates HKDC1 from VDAC1 and induces mitochondrial dysfunction and apoptosis in ENKTL cells. SNK6 cells were treated by control (CTL), Tf-D-HKN15, Tf-D-HKC15, or Tf-D-HKC8 peptide (0.5 µM) for 6 h, and the cells were used for biomedical analysis. **a** Protein quantitation by western blots, *n* = 5. **b** Representative western blotting bands for **a**. **c** Representative western blotting bands for IP/WB. **d** Protein quantitation for IP/WB, *n* = 5. **e** Hexokinase activity, *n* = 5. **f** Mitochondrial membrane potential (∆Ψm), *n* = 5. **g** Intracellular ATP level, *n* = 5. **h** Apoptosis rate by TUNEL assay, *n* = 5. **i** Representative pictures for **h**. *P < 0.05, vs CTL group; ^¶^*P* < 0.05, vs Tf-D-HKC15 group. Results were expressed as mean ± SEM.

We first evaluated the effect of HKDC1-based peptides on the association of HKDC1 and VDAC1. The results showed that protein levels of HKDC1 and VDAC1 had no changes based on different treatments (see Fig. 4a, b), while peptides Tf-D-HKC15 and Tf-D-HKC8 decreased association of HKDC1 with VDAC1 to 68% and 24%, respectively, and peptide Tf-D-HKN15 had no effect (see Fig. 4c, d). We also conducted IP/WB to evaluate the potential effect of HKC8 on HKDC1/VDAC1 interaction in different cells, including normal HMECs cells and two kinds of cancer cell lines, HANK1 and SW480. The results showed that HKC8 decreased association of HKDC1 with VDAC1 to 34% and 67% in HANK1 and SW480 cells, respectively, compared with the CTL group, while it had little effect on HMECs (see Fig S3). This may be partly because the normal cells have much lower levels of TfR expression, resulting in Tf-D-HKC8’s poor delivery efficiency. In addition, the normal cells have much less dependency on HKDC1/VDAC association-mediated normal metabolism compared with cancer cell lines. Furthermore, the peptides had no effect on the total HK activity (see Fig. 4e), while peptides Tf-D-HKC15 and Tf-D-HKC8 decreased mitochondrial membrane potential (ΔΨm) to 68% and 24%, respectively (see Fig. 4f), and decreased intracellular ATP generation to 61% and 30%, respectively (see Fig. 4g). Finally, we measured peptide-mediated apoptosis in SNK6 cells. The results showed that peptides Tf-D-HKC15 and Tf-D-HKC8 increased apoptosis rate by 2.91- and 11.4-fold, respectively, compared with the CTL group; and again, peptide Tf-D-HKN15 had no effect (see Fig. 4h, i). Our results indicate that peptide Tf-D-HKC8 dissociates HKDC1 with VDAC1 and induces mitochondrial function and apoptosis in ENKTL cells.

### Peptide Tf-D-HKC8 inhibits tumor growth in ENKTL cells

**Fig. 5:**
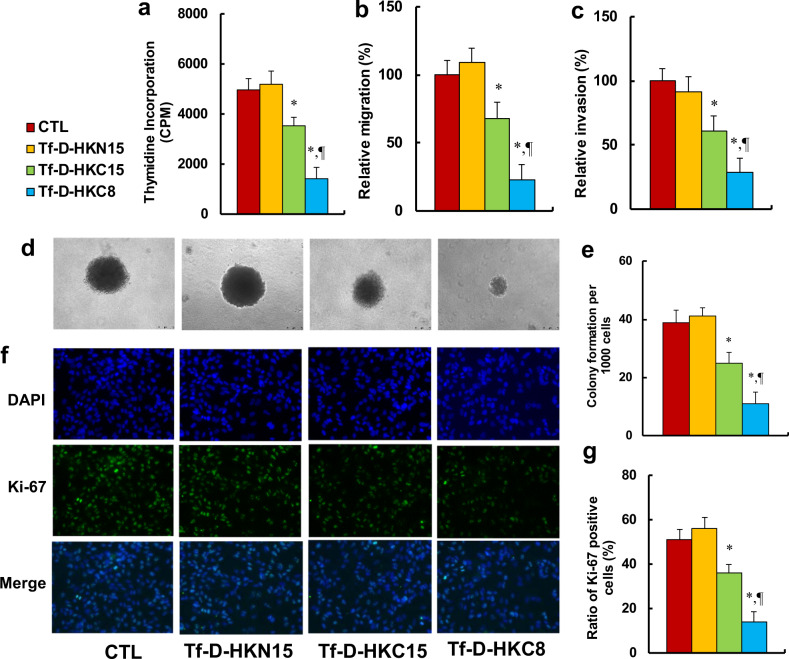
Peptide Tf-D-HKC8 inhibits tumor growth in ENKTL cells. SNK6 cells were treated with control (CTL), Tf-D-HKN15, Tf-D-HKC15, or Tf-D-HKC8 peptide (0.5 µM) for 6 h and the cells were used for biomedical analysis. **a** Cell proliferation analysis by thymidine incorporation, *n* = 5. **b** Cell migration assay, *n* = 5. **c** Cell invasion assay, *n* = 5. **d** Representative pictures for colony formation. **e** Colony formation assay in soft agar, *n* = 5. **f** Representative picture for Ki-67 staining. **g** Quantitation of Ki-67 positive cells, *n* = 3. *P < 0.05, vs CTL group; ^¶^*P* < 0.05, vs Tf-D-HKC15 group. Results were expressed as mean ± SEM.

We investigated the potential effects of HKDC1-based peptides on tumor growth in ENKTL cells. The results showed that peptides Tf-D-HKC15 and Tf-D-HKC8 decreased thymidine incorporation to 71% and 28%, respectively, while peptide Tf-D-HKN15 showed no effect (see Fig. 5a). We then measured the effect of HKDC1-based peptides on metastasis in SNK6 cells. The results showed that peptides Tf-D-HKC15 and Tf-D-HKC8 decreased cell migration to 66% and 23%, respectively (see Fig. 5b), and decreased cell invasion to 61% and 29%, respectively (see Fig. 5c). We then evaluated colony formation, and our findings showed that peptides Tf-D-HKC15 and Tf-D-HKC8 decreased colony formation to 64% and 28%, respectively (see Fig. 5d, e). Finally, we evaluated the effect of peptides on cell proliferation by quantitation of Ki-67 positive cells. The results showed that peptides Tf-D-HKC15 and Tf-D-HKC8 decreased cell proliferation to 70% and 27%, respectively; again, peptide Tf-D-HKN15 showed no effect (see Fig. 5f, g). Our results indicate that peptide Tf-D-HKC8 inhibits tumor growth in ENKTL cells.

### Peptide Tf-D-HKC8 inhibits tumor growth and EBV replication in ENKTL xenograft mouse models

**Fig. 6:**
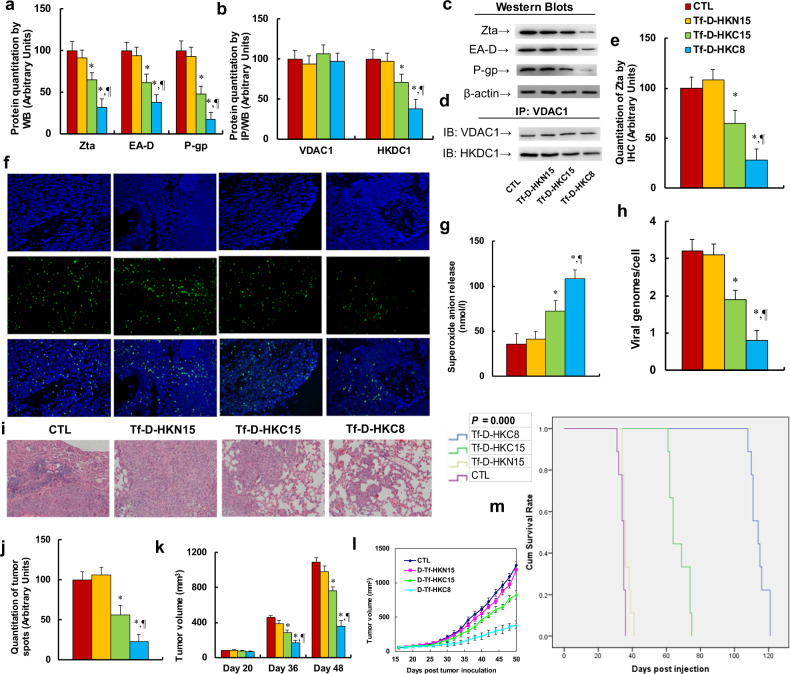
Peptide Tf-D-HKC8 inhibits tumor growth and EBV replication in ENKTL xenograft mouse models. A total of 1 × 10^5^ of SNK6 cells were injected subcutaneously along the mouse flank for xenograft tumor growth. On day 18 after tumor inoculation, the mice were randomly divided into four groups and treated by control (CTL), Tf-D-HKN15, Tf-D-HKC15, or Tf-D-HKC8 peptide (0.5 µM) every 2 days. The tumor tissues were isolated for biomedical analysis on day 50. **a** Protein quantitation by western blots, *n* = 5. **b** Protein quantitation for IP/WB, *n* = 5. **c** Representative western blotting bands for **a**. **d** Representative western blotting bands of IP/WB for **b**. **e** Quantitation of Zta by IHC, *n* = 3. **f** Representative pictures of IHC for **e**. **g** Superoxide anion release, *n* = 5. **h** EBV viral genomes/cell by qPCR, *n* = 4. **i** Representative pictures for H&E staining. **j** Quantitated tumor spots for **i**, *n* = 5. **k** Quantitated tumor volume on day 20, 36, and 48, *n* = 5. **l** Quantitated curves for tumor volumes, *n* = 5. **m** Kaplan–Meier analysis comparing survival of mice between each treatment group, *P* value represents log-rank Mantel–Cox test result, *n* = 9. *P < 0.05, vs CTL group; ^¶^*P* < 0.05, vs Tf-D-HKC15 group. Results were expressed as mean ± SEM.

We investigated the potential effect of HKDC1-based peptides on tumor growth and EBV replication through in vivo xenograft mouse model. We first measured the protein levels of those genes. The results showed that peptides Tf-D-HKC15 and Tf-D-HKC8 decreased EBV Zta protein levels to 65% and 32%, respectively; decreased EBV EA-D protein to 61% and 38%, respectively; and decreased P-gp protein to 48% and 17%, respectively, (see Fig. 6a, c). We then measured the association of HKDC1 and VDAC1 using IP/WB techniques. The results showed that peptides Tf-D-HKC15 and Tf-D-HKC8 decreased HKDC1 binding with VDAC1 to 71% and 38%, respectively, (see Fig. 6b, d). We also evaluated the gene expression of Zta on tumor tissues using IHC techniques. The results showed that peptides Tf-D-HKC15 and Tf-D-HKC8 decreased Zta gene expression to 64% and 28%, respectively, (see Fig. 6e, f). We then evaluated the effect of peptides on superoxide anion (O_2_^.−^) release and EBV genome replication. The results showed that peptides Tf-D-HKC15 and Tf-D-HKC8 increased superoxide anion release to 203% and 297%, respectively, (see Fig. 6g), while they decreased EBV genome replication to 59% and 25%, respectively, (see Fig. 6h). We further evaluated the effect of peptides on tumor growth using H&E staining. The results showed that peptides Tf-D-HKC15 and Tf-D-HKC8 decreased tumor growth to 56% and 23%, respectively, (see Fig. 6i, j). We finally evaluated the effect of peptides on the tumor volume changes. Our findings showed that peptides Tf-D-HKC15 and Tf-D-HKC8 decreased tumor volumes to 63% and 36% on day 36, respectively, while they decreased tumor volumes to 70% and 32% on day 48, respectively; again, peptide Tf-D-HKN15 had no effect (see Fig. 6k, l). Finally, we measured the effect of peptides on mouse survival rate using Kaplan–Meier analysis (see Fig. 6m). We found that peptide Tf-D-HKN15 had little effect on mouse survival, while peptides Tf-D-HKC15 and Tf-D-HKC8 significantly increased mouse survival to 197% and 335% compared with the CTL group, respectively. Our results indicate that peptide Tf-D-HKC8 inhibits ENKTL tumor growth and EBV replication in in vivo xenograft mouse models.

## Discussion

In this study, we have identified a novel antitumor target for ENKTL treatment based on the last eight aa of HKDC1 at the C-terminal. We show that delivery of Tf-D-HKC8 peptide dissociates HKDC1 from VDAC1, induces mitochondrial dysfunction and oxidative stress, and subsequently suppresses tumor growth. Interestingly, peptide Tf-D-HKC8-induced ROS overgeneration significantly suppresses EBV replication and P-gp expression, making the peptide highly susceptible to EBV-associated ENKTL tumors.

### Tf-D-HKC8 peptide-induced suppression of EBV and P-gp

We show that peptide Tf-D-HKC8 induces significant cell death in different kinds of cancer cells, and EBV-positive ENKTL cells [31] are more susceptible to Tf-D-HKC8 peptide-induced cytotoxicity than EBV-negative cancer cells. Tf-D-HKC8 peptide dissociates HKDC1 from VDAC1 and induces significant ROS overgeneration, subsequently resulting in EBV DNA damage and P-gp suppression. This is consistent with our previous finding that ROS overgeneration suppresses EBV-positive ENKTL cells [7]. P-gp is a membrane transporter coded by multiple drug resistance 1 gene that excretes drugs from the cytoplasm, resulting in high resistance to chemotherapy [9]. It has been reported that latent EBV infection triggers P-gp expression [10, 11], although the exact mechanism for upregulated P-gp expression in ENKTL remains unclear. Our results show that Tf-D-HKC8 peptide-induced ROS overgeneration results in EBV DNA damage and P-gp suppression, making it very effective to kill EBV-positive ENKTL cells. It has been reported that EBV infection causes hypoxic conditions, induces intracellular ROS generation, and activates the STAT1 signaling pathway. Subsequently, phosphorylated STAT1 promotes P-gp expression; thus, it can be concluded that ROS generation induces P-gp expression [14]. Interestingly, our results show that Tf-D-HKC8 peptide-induced dissociation of HKDC1 from VDAC1 induces interrupted glucose metabolism and ROS overgeneration, resulting in EBV DNA breaks and eventually suppressing P-gp expression by interrupting the potential driving force triggered by EBV DNA. These two findings may potentially be consistent with one another, providing us with a new strategy for EBV-positive ENKTL treatment by triggering ROS generation and the anti-metabolism signaling pathway.

### HKDC1-based novel strategy for ENKTL treatment

HKDC1 is a novel HK isoform that is involved in glucose metastasis [15, 18, 19]. Upregulated expression of HK enzymes has been reported in many different tumors [32]. Our results show that HKDC1 is highly expressed in ENKTL and is involved in cell proliferation and tumor growth [20, 23]. Furthermore, the peptide for the last eight aa of HKDC1 at the C-terminal (HKC8) can significantly suppress the association of HKDC1 with VDAC1, interrupt glucose metabolism, induce apoptosis, and ROS overgeneration, and subsequently suppress EBV replication and P-gp expression. HKDC1 could be a novel potential therapeutic target for antitumor drug development [20–22, 33]. ENKTL is highly resistant to current multidrug chemotherapies with a poor prognosis [6, 7]. Many potential risk factors for ENKTL have been described [34], and EBV infection is considered to be the strongest one [31]. Currently, L-asparaginase-based antimetabolic chemotherapy remains the primary treatment for relapsed/refractory ENKTL with significant off-target effects on killing normal cells [13]. Our results show that peptide Tf-D-HKC8-induced ROS overgeneration induces EBV DNA damage directly and suppresses P-gp expression. Furthermore, peptide Tf-D-HKC8 specifically inhibits HKDC1 binding activity, and HKDC1 is highly expressed in tumor cells instead of normal cells, this allows minimal cytotoxicity of peptides to normal cells. Taken together, HKDC1-based peptides could be a novel therapeutic target specific for EBV-positive ENKTL treatment. On the other hand, peptide HKC8 has many obstacles for application of ENKTL clinical treatment. This includes, but is not limited to, short half-life time, limited delivery efficiency using cell-penetrating sequence, inability to be given through oral administration, etc. Thus, the development of small molecules mimicking the effect of HKC8 is now under our investigation [35–37] and could bring us new hope for ENKTL treatment in the future.

## Conclusions

Peptide HKC8 dissociates HKDC1 from VDAC1, interrupts glucose metabolism, induces ROS overgeneration, resulting ENKTL cell apoptosis, EBV DNA damage, and P-gp suppression. HKC8 is a novel therapeutic target for ENKTL antitumor drug development.

## Supplementary information

Supplemental Information Article

Supplemental Information Figure S1

Supplemental Information Figure S2

Supplemental Information Figure S3
