# Supplementary material for: HKDC1 C-terminal based peptides inhibit extranodal natural killer/T-cell lymphoma by modulation of mitochondrial function and EBV suppression
Source: Leukemia. 2020 Mar 23;34(10):2736–48. doi: 10.1038/s41375-020-0801-5 (PMC7515829; doi:10.1038/s41375-020-0801-5)
Supplement: Supplementary file 1 — Supplemental Information Article [file 41375_2020_801_MOESM1_ESM.docx]

**HKDC1 C-Terminal Based Peptides Inhibit Extranodal Natural Killer/T-Cell Lymphoma by Modulation of Mitochondrial Function and EBV Suppression**

Qi Chen, Jia Feng, Jinhu Wu, Zhendong Yu, Wei Zhang, Yonggang Chen,

Paul Yao, Hongyu Zhang

**Supplementary Materials**

**Data S1.** MATERIALS AND METHODS

**Reagents and materials**. Written consent was obtained from participants and the mononuclear cells (MNCs) from either healthy or ENKTL patients were isolated from peripheral blood using Lymphoprep^TM^ reagents (#07861). The ENKTL cell lines, including HANK1, NK92, SNT8 and SNK6 cells, were purchased from ATCC and cultured in RPMI 1640 medium containing 2mmol/l glutamine supplemented with 100 U/ml penicillin, 100 μg/ml streptomycin, 10% human serum and 1000 U/ml recombinant human IL-2. The primary HMECs and tumor cell lines, including MCF7, MDA-MB-231, SW480, SW620, Hela, and HepG2 cells were obtained from ATCC and cultured in DMEM supplemented with 10% FBS and antibiotics. All cells were maintained in a humidified incubator with 5% CO_2_ at 37°C.

Antibodies for β-actin (sc-47778), EBV Ea-D (sc-58121), EBV ZEBRA (BZ1, sc-53904), Ki-67 (sc-101861) and VDAC1 (sc-390996) were obtained from Santa Cruz Biotechnology. Antibodies for HK2 (ab104836), H2AX (ab20669), γH2AX (ab2893) and P-gp (ab129450) were obtained from Abcam. The antibody for HKDC1 (NBP1-82108) was obtained from Novus Biologicals. 3-nitrotyrosine (3-NT) was measured by 3-Nitrotyrosine ELISA Kit (ab116691 from Abcam). Nuclear extracts were prepared using the NE-PER Nuclear and Cytoplasmic Extraction Reagents Kit (Pierce Biotechnology). Protein concentration was measured using the Coomassie Protein Assay Kit (Pierce Biotechnology) per manufacturers’ instructions.

**Preparation of knockdown lentivirus.** The lentivirus for stable HKDC1 knockdown was prepared in our lab (1). The stable knockdown cells for HK2 and related non-target control (CTL) were prepared through infection of tumor cell lines (e.g. SNK6) using shRNA lentivirus particles from Sigma for human HK2 (SHCLNV-NM_000189), or non-target control (SHC216V). The positive knockdown cells were selected using 10μg/ml of puromycin, and the stable knockdown cell line was confirmed by real time PCR based on an mRNA decrease of more than 65% compared to the control group (see primers in Table S1) (1, 2).

**Preparation of expression lentivirus**. The lentivirus for HKDC1 expression was generated in our lab previously (1, 2). In order to prepare SOD2, HK2 and HKDC1 c-terminal deletion (HKDC1-Δc8a) expression constructs, the SOD2, HK2 and HKDC1-Δc8a was amplified from full length cDNA of SOD2, HK2 and HKDC1 (obtained from Open Biosystems) and subcloned into the pLVX-Puro vector (from Clontech) using the following primers with the introduction of underlined restriction sites: SOD2 forward primer: 5’- gtac- ctcgag- atg ttg agc cgg gca gtg tgc -3’ (Xho1) and human SOD2 reverse primer: 5’- gtac- tctaga- tta ctt ttt gca agc cat gta -3’ (Xba1); HK2 forward primer: 5’- ATCG- GAATTC- atg att gcc tcg cat ctg ctt - 3’ (EcoR1) and HK2 reverse primer: 5’- ATCG- GGATCC- cta tcg ctg tcc agc ctc acg - 3’ (BamH I); HKDC1-Δc8a forward primer: 5’- ATCG- CTCGAG- atg ttt gcg gtc cac ttg atg -3’ (Xho I) and HKDC1-Δc8a reverse primer: 5’- ATCG- GGATCC- cta cct ctt ggc cac agc agt gat - 3’ (BamH I). The virus for SOD2, HK2, HKDC1-Δc8a or empty control (CTL) was expressed by Lenti-X™ Lentiviral Expression Systems (from Clontech), and this lentivirus was used for generation of stable tumor cell lines for overexpression of SOD2, HK2 or HKDC1-Δc8a (1, 2).

**Peptide synthesis and preparation**. The HKDC1-based peptides were synthesized by GL Biochem (Shanghai, China) with a level of > 85% purity. The peptides were dissolved in DMSO (dimethyl sulfoxide) and stored in aliquots at -20°C. Peptide concentration was determined by UV spectrophotometry at 280nm and calculated based on amino acid composition. The final concentration of DMSO used in this study was controlled no more than 0.5%. Detailed information on the synthesized peptides is shown in Table 1. The cell-penetrating sequence is underlined, the HKDC1-targeting sequences are in italics, and amino acids in the D-configuration are marked in bold (3).

**Hexokinase activity assay.** Total HK activity from cell lysates was measured as the glucose phosphorylating capacity of whole cell extracts using a standard G6PDH-coupled assay (4, 5). The glucose and ATP-dependent reduction of NADP was monitored by a 96-well microplate reader at 340nm in the presence of excess G6PDH. All assays (final assay mixture composition: 1 U/ml G6PDH, 0.5mg/ml NADP, 6.7mM ATP, 7.7mM MgCl2, 4.0mM Glucose, 45mM KCl, 1mM NaH2PO4, 10.6mM monothioglycerol, 0.01% Triton X-100, 0.5mM EDTA, and 42 mM Tris HCl, pH 8.5) were performed at 25°C under conditions of linear HK-limited NADPH formation. Total HK activity was normalized for cellular protein content and was expressed in enzyme activity units corresponding to the glucose phosphorylation rate in micromoles per minute (6).

**RT reaction and real-time quantitative PCR.** Total RNA from treated cells was extracted using the RNeasy Micro Kit (Qiagen), and the RNA was reverse transcribed using an Omniscript RT kit (Qiagen). All the primers were designed using Primer 3 Plus software with the Tm at 60°C, primer size of 21bp, and the product length in the range of 140-160bp (see Table S1). The primers were validated with the amplification efficiency in the range of 1.9-2.1, and the amplified products were confirmed with agarose gel. Real-time quantitative PCR was run on iCycler iQ (Bio-Rad) using the Quantitect SYBR green PCR kit (Qiagen). The PCR was performed by denaturing at 95°C for 8 min followed by 45 cycles of denaturation at 95°C, annealing at 60°C, and extension at 72°C for 10s, respectively. 1 µl of each cDNA was used to measure target genes. β-actin was used as the housekeeping gene for transcript normalization, and the mean values were used to calculate relative transcript levels with the ^ΔΔ^CT method per instructions from Qiagen. In brief, the amplified transcripts were quantified by the comparative threshold cycle method using β-actin as a normalizer. Fold changes in gene mRNA expression were calculated as 2^−ΔΔCT^ with CT = threshold cycle, ΔCT=CT (target gene)-CT(β-actin), and the ΔΔCT =ΔCT (experimental)-ΔCT (reference) (7, 8).

**Immunoprecipitation (IP) and Western Blotting (WB).** Cell lysates were pre-cleared by pre-immune IgG plus Protein A agarose beads for 2 hours, and the supernatants were immunoprecipitated by the indicated antibodies and a 50% slurry of Protein A Agarose beads overnight at 4˚C (9). After washing with buffer containing 50 mM Tris, pH 7.5, 150 mM NaCl, 1% NP-40, and 0.5% deoxycholate with protease inhibitors, proteins were released, separated on 10% SDS-PAGE gels, blotted by primary antibodies, and then simultaneously incubated with the differentially labeled species-specific secondary antibodies, anti-RABBIT IRDye™ 800CW (green) and anti-MOUSE (or goat) ALEXA680 (red). Membranes were scanned and quantitated by the ODYSSEY Infrared Imaging System (LI-COR, NE) (10).

**Immunostaining**. The treated SNK6 cells were transferred to cover slips coated with 0.1% gelatin, fixed by 3.7% formaldehyde at 37ºC for 15 min, permeabilized by 1% BSA+0.2% Triton X-100 in PBS for 1 hour, and then blotted with 40μg/ml (dilute 1:50) of Ki-67 (MIB-1) mouse monoclonal antibody for 2 hours. The cells were then washed three times and the FITC labeled anti-mouse secondary antibody (1:100) was added for blotting for another 1 hour. After thorough washing, the slides were visualized and photographed, the nuclei of cells were stained with 4’,6-diamidino-2-phenylindole dihydrochloride (DAPI, #D9542, from Sigma), and the positive Ki-67 cells were quantitated.

**[^3^H]-deoxyglucose uptake.** 1x106 treated cells were suspended and rinsed with PBS 3 times and then incubated with 1ml of PBS containing 1.0uCi 3H-deoxyglucose for 5 min at 37°C. Cells were washed with cold PBS 3 times and solubilized in 1ml of 1M NaOH for 60 min at 37°C. They were then neutralized with an equal volume of 1 M HCl and counted in 10 ml scintillation mixture and the final results were normalized by protein level.

**Measurement of ROS generation.** Treated cells were seeded in a 24-well plate and incubated with 10μM CM-H2DCFDA (Invitrogen) for 45 min at 37°C, and then the intracellular formation of reactive oxygen species (ROS) was measured at excitation/emission wavelengths of 485/530nm using a FLx800 microplate fluorescence reader (Bio-Tek). The data was normalized as arbitrary units (8, 11).

**Measurement of DNA breaks**. 8-OHdG formation was measured using an OxiSelect™ Oxidative DNA Damage ELISA Kit (Cat No. STA320, from Cell Biolabs Inc.) per manufacturers’ instructions. The formation of γH2AX was measured from nuclear extracts by western blotting using H2AX as the input control (8).

**Measurement of apoptosis and cell death.** Apoptosis was evaluated by TUNEL assay using the In Situ Cell Death Detection Kit™ (Roche). Cells were fixed in 4% paraformaldehyde and labeled with TUNEL reagents. Stained cells were photographed by a fluorescence microscope (11). For cell death analysis, the propidium iodide (PI) staining was performed by addition of 6.25 µg/ml of PI to the cells, and cell death was immediately evaluated by FACS analysis (3). Different concentrations of peptides were used for treatment of either adhesion (12 hours) or suspension (6 hours) cells, and the half-maximal cell death activity (IC_50_) values (µM) were then calculated based on the cell death data obtained from PI/FCAS analysis (3).

**Measurement of mitochondrial function.** Intracellular ATP level was determined using the luciferin/luciferase-induced bioluminescence system. An ATP standard curve was generated at concentrations of 10^-12^-10^-3^M, and intracellular ATP levels were calculated and expressed as nmol/mg protein. Mitochondrial membrane potential (Δψm) was measured using TMRE (from Molecular Probes T-669) staining. A 600μM T-669 stock solution was prepared using DMSO. Cells were grown on coverslips and immersed in 600nM TMRE for 20 min at 37°C to load them with dye. The labeling medium was then aspirated and the cells were immersed in 150nM TMRE to maintain an equal distribution of the fluorophore. The coverslips were mounted with live cells onto confocal microscopes to image the cells using 548nm excitation/573nm emission filters, and the intensity of TMRE fluorescence was measured using Image J software. Data from 10-20 cells were collected for each experimental condition and mean values of fluorescence intensity ± SEM were calculated (12).

**Detection of EBV copy number.** Genomic DNA was extracted from treated SNK-6 cells or SNK-6 tumor tissue in mice using a QIAamp DNA Mini Kit (Qiagen). The EBV DNA copy number was measured through qPCR using 50ng of total DNA with EBV BMRF1 primers (see Table 1), and the results were normalized using cellular β-actin (primers see Table 1) as an internal control (13, 14). The Namalwa cell line, which contains 2 EBV viral genome copies, was used as a standard to prepare calibration curves for both EBV BMRF1 and β-actin genes, and the EBV viral load was presented as the number of viral genomes per cell (15, 16).

**DNA synthesis by [^3^H]-thymidine incorporation**. Cell proliferation was evaluated as the rate of DNA synthesis by [^3^H]-methylthymidine incorporation (17). Cells were pooled in 24-well plates until they reached 80% confluence and then the indicated chemicals were added and incubated for 24 hours. At the end of the treatment, cells were incubated with serum-free media containing ^3^H-methylthymidine (0.5 µCi/well) for 2 hours and then washed twice with PBS. Cellular DNA was precipitated using 10% trichloroacetic acid and solubilized with 0.4M NaOH (0.5 ml/well). Incorporation of ^3^H-methylthymidine into the DNA was measured in a scintillation counter and was determined as counts per minute (CPM) (8).

**Colony formation in soft agar**. This assay is a method for evaluating the ability of individual cell lines to grow in an anchorage-independent manner. Cells were resuspended in DMEM containing 5% FBS with 0.3% agarose and layered on top of 0.5% agarose in DMEM on 60-mm plates. 1000 cells were seeded in 60mm soft agar dishes for 30 days. The dishes were examined twice per week, and colonies that grew beyond 50mm in diameter were scored as positive. Each experiment was done in quadruplicate (8).

**Migration and invasion assays**. Cell migration and invasion assays were performed in 24-well chemotaxis plates with an 8μm polycarbonate filter membrane. The plates were coated with 20μg Matrigel for invasion assays and uncoated for migration assays. Invasion and migration were expressed as the number of migrated cells bound per microscopic field and averaged from at least four fields per assay in at least 4 experiments (18, 19).

**In vivo mouse experiments**. Balb/c athymic nude male mice (6 weeks old) were obtained from the Guangdong Medical Animal Center. All procedures involving mice were conducted in accordance with NIH regulations concerning the use and care of experimental animals and were approved by the Institutional Animal Care and Use Committee of Peking University Shenzhen Hospital. The protocol was pre-established, and the animal group was randomly selected and the experiments were conducted by double blinding.

*In vivo mouse protocol*. To measure the malignant SNK6 xenograft growth, the 100µl of 1x10^5^ SNK6 cells in PBS were mixed with 100µl growth factor-reduced basement membrane matrix (Matrigel™, BD Biosciences) resulting in 200µl of solution that was injected subcutaneously along the mouse flank at each intended tumor site. Tumor size was measured using a digital caliper and volume was calculated, and mice were randomly divided into 4 groups (n=9 per group) when the tumor volume reached 50-100 mm^3^ on day ~18 after tumor inoculation: Group 1 (CTL) was intratumorally injected with HBSS buffer (5.33 mM KCl, 0.44 mM KH2PO4, 138 mM NaCl, 4 mM NaHCO3, 0.3 mM Na2HPO4, and 5.6 mM glucose, pH 7.3) containing 0.05% DMSO; Group 2 (Tf-D-HKN15) received HBSS buffer containing 0.05% DMSO with 50 µM Tf-D-HKN15 peptides; Group 3 (Tf-D-HKC15) received HBSS buffer containing 0.05% DMSO with 50 µM Tf-D-HKC15 peptides; Group 4 (Tf-D-HKC8) received HBSS buffer containing 0.05% DMSO with 50 µM Tf-D-HKC8 peptides. The xenografts were injected (two points, 20 µL per tumor) every two days. Beginning on the day of inoculation, mouse weight and tumor volume were monitored every 2 days. Mice were monitored for changes in body weight and sacrificed when values fell below 20% of their initial weight, the survival curve was calculated and the final tumor tissues were isolated for biomedical analysis.

*Biomedical analysis of tumor tissues*. Part of the tumor tissues were fixed in 4% buffered formaldehyde, paraffin embedded, and sectioned to 4mm thickness. They were then either processed for immunohistochemistry (IHC) or histopathological analyses were performed with H&E staining. Images were taken using a Carl Zeiss MIRAX MIDI slide scanner, and analyses were performed using a 3DHISTECH Pannoramic Viewer. Part of the tumor tissues were isolated for *in vivo* monitoring of superoxide anion release, gene expression were measured through real time PCR for mRNA and Western Blotting for protein levels, the copies of the EBV genome were measured by real time PCR, and the binding of HKDC1 and VDAC1 was evaluated by IP/WB (8).

**Immunohistochemistry (IHC).** The tumor tissue slides were first fixed by 3.7% formaldehyde solution, permeabilized by 1% BSA+0.2% Triton X-100 in PBS, and then blotted by 40μg/ml of mouse antibody for Zta for 2 hours. After another 1 hour of blotting by FITC labeled anti-mouse secondary antibody, the slides were visualized and photographed, and the protein expression (60 cells in each group) were quantitated by Image J. software (2).

**In vivo superoxide release analysis.** Superoxide anion (O_2_^.-^) release from tumor tissues was determined using a luminol-EDTA-Fe enhanced chemiluminescence (CL) system supplemented with DMSO-TBAC (Dimethyl sulfoxide-tetrabutyl-ammonium chloride) solution for extraction of released O_2_^.-^ from tissues, as described previously (11). Superoxide levels were calculated from the standard curve generated by the xanthine/xanthine oxidase reaction (8).

**Statistical analysis.** The data was given as mean ± SEM; all of the experiments were performed at least in quadruplicate unless otherwise indicated. One-way ANOVA followed by the Bonferroni post hoc test was used to determine statistical significance of different groups. The mouse survival curve was determined through Kaplan-Meier survival analysis using SPSS 22 software, and a *P* value < 0.05 was considered significant (8).

REFERENCES

1. Chen X, Lv Y, Sun Y, Zhang H, Xie W, Zhong L, Chen Q, Li M, Li L, Feng J, et al. PGC1beta Regulates Breast Tumor Growth and Metastasis by SREBP1-Mediated HKDC1 Expression. Front Oncol. 2019;9(290.

2. Zhang H, Li L, Chen Q, Li M, Feng J, Sun Y, Zhao R, Zhu Y, Lv Y, Zhu Z, et al. PGC1beta regulates multiple myeloma tumor growth through LDHA-mediated glycolytic metabolism. Mol Oncol. 2018;12(9):1579-95.

3. Shteinfer-Kuzmine A, Amsalem Z, Arif T, Zooravlov A, and Shoshan-Barmatz V. Selective induction of cancer cell death by VDAC1-based peptides and their potential use in cancer therapy. Mol Oncol. 2018;12(7):1077-103.

4. Bryson JM, Coy PE, Gottlob K, Hay N, and Robey RB. Increased hexokinase activity, of either ectopic or endogenous origin, protects renal epithelial cells against acute oxidant-induced cell death. J Biol Chem. 2002;277(13):11392-400.

5. Majewski N, Nogueira V, Bhaskar P, Coy PE, Skeen JE, Gottlob K, Chandel NS, Thompson CB, Robey RB, and Hay N. Hexokinase-mitochondria interaction mediated by Akt is required to inhibit apoptosis in the presence or absence of Bax and Bak. Mol Cell. 2004;16(5):819-30.

6. Kroschewski H, Ortner S, Steipe B, Scheiner O, Wiedermann G, and Duchene M. Differences in substrate specificity and kinetic properties of the recombinant hexokinases HXK1 and HXK2 from Entamoeba histolytica. Mol Biochem Parasitol. 2000;105(1):71-80.

7. Zou Y, Lu Q, Zheng D, Chu Z, Liu Z, Chen H, Ruan Q, Ge X, Zhang Z, Wang X, et al. Prenatal levonorgestrel exposure induces autism-like behavior in offspring through ERbeta suppression in the amygdala. Mol Autism. 2017;8(46.

8. Zhang H, Li L, Li M, Huang X, Xie W, Xiang W, and Yao P. Combination of betulinic acid and chidamide inhibits acute myeloid leukemia by suppression of the HIF1alpha pathway and generation of reactive oxygen species. Oncotarget. 2017;8(55):94743-58.

9. Metivier R, Penot G, Hubner MR, Reid G, Brand H, Kos M, and Gannon F. Estrogen receptor-alpha directs ordered, cyclical, and combinatorial recruitment of cofactors on a natural target promoter. Cell. 2003;115(6):751-63.

10. Ceradini DJ, Yao D, Grogan RH, Callaghan MJ, Edelstein D, Brownlee M, and Gurtner GC. Decreasing intracellular superoxide corrects defective ischemia-induced new vessel formation in diabetic mice. J Biol Chem. 2008;283(16):10930-8.

11. Yao D, Shi W, Gou Y, Zhou X, Yee Aw T, Zhou Y, and Liu Z. Fatty acid-mediated intracellular iron translocation: a synergistic mechanism of oxidative injury. Free Radic Biol Med. 2005;39(10):1385-98.

12. Kong D, Zhan Y, Liu Z, Ding T, Li M, Yu H, Zhang L, Li H, Luo A, Zhang D, et al. SIRT1-mediated ERbeta suppression in the endothelium contributes to vascular aging. Aging Cell. 2016.

13. Verma D, Thompson J, and Swaminathan S. Spironolactone blocks Epstein-Barr virus production by inhibiting EBV SM protein function. Proc Natl Acad Sci U S A. 2016;113(13):3609-14.

14. Zuo L, Yu H, Liu L, Tang Y, Wu H, Yang J, Zhu M, Du S, Zhao L, Cao L, et al. The copy number of Epstein-Barr virus latent genome correlates with the oncogenicity by the activation level of LMP1 and NF-kappaB. Oncotarget. 2015;6(38):41033-44.

15. Hui KF, and Chiang AK. Suberoylanilide hydroxamic acid induces viral lytic cycle in Epstein-Barr virus-positive epithelial malignancies and mediates enhanced cell death. Int J Cancer. 2010;126(10):2479-89.

16. Rose C, Green M, Webber S, Kingsley L, Day R, Watkins S, Reyes J, and Rowe D. Detection of Epstein-Barr virus genomes in peripheral blood B cells from solid-organ transplant recipients by fluorescence in situ hybridization. J Clin Microbiol. 2002;40(7):2533-44.

17. Somasundaram K, and El-Deiry WS. Inhibition of p53-mediated transactivation and cell cycle arrest by E1A through its p300/CBP-interacting region. Oncogene. 1997;14(9):1047-57.

18. Han HJ, Russo J, Kohwi Y, and Kohwi-Shigematsu T. SATB1 reprogrammes gene expression to promote breast tumour growth and metastasis. Nature. 2008;452(7184):187-93.

19. Yu OM, Benitez JA, Plouffe SW, Ryback D, Klein A, Smith J, Greenbaum J, Delatte B, Rao A, Guan KL, et al. YAP and MRTF-A, transcriptional co-activators of RhoA-mediated gene expression, are critical for glioblastoma tumorigenicity. Oncogene. 2018.

**Table S1. Sequences of primers for the real time quantitative PCR (qPCR)**

| Gene | Species | Analysis | Forward primer (5'→3') | Reverse primer (5'→3') |
| --- | --- | --- | --- | --- |
| β-actin | Human | mRNA | gatgcagaaggagatcactgc | atactcctgcttgctgatcca |
| BZLF1 | EBV | mRNA | gggggataatggagtcaacat | tagcgtcccaaacataaatgc |
| BMRF1 | EBV | mRNA | tcctgtccaagtgctatgacc | gggagacctcgaagctgatta |
| β-actin | Human | Genome | ctggacttcgagcaagagatg | aggaaggaaggctggaagagt |
| BMRF1 | EBV | Genome | ccgtcctgtccaagtgctat | gggagacctcgaagctgatta |
| HK1 | Human | mRNA | cctgcatctctgacttcttgg | acgcagtctgttgccttaaaa |
| HK2 | Human | mRNA | gatttcaccaagcgtggacta | aagccctaagtgttgcaggat |
| HK3 | Human | mRNA | agggtttcaaggcatcagact | tcctcatagccacaggacatc |
| HK4 | Human | mRNA | tatcaaacggagaggggactt | cattctgcatctcctccatgt |
| HKDC1 | Human | mRNA | acgagtttgacctggacattg | ccatctcgatgttcctcatgt |
| ABCB1 | Human | mRNA | atagacagccgcagtcaagaa | gctactgtctttcctcgctca |

FIGURE LEGENDS

**Figure S1. Peptide delivery efficiency assay using ^125^I-labelled peptides.** Peptides Tf-D-HKC8 and Tf-D-HKC15 were synthesized and labelled by ^125^I isotope on tyrosine (Y) residue. 0.5µM each of peptide was incubated with SNK6 cells for 6 hours, then both culture media and SNK6 cells were harvested for radioactivity assay using Liquid Scintillation Counter. n=4. *, *P*<0.05, vs Intracellular group. Results were expressed as mean ± SEM.

**Figure S2. SOD2 overexpression restores peptide Tf-D-HKC8-mediated EBV suppression in NKTCL cells.** SNK6 cells were treated by either control (CTL), 0.5µM of Tf-D-HKC8 peptide (Tf-D-HKC8) or 0.5µM of Tf-D-HKC8 peptide with SOD2 expression lentivirus (Tf-D-HKC8/SOD2) for 2 hours, and the cells were harvested for biomedical analysis. (a) ROS formation assay, n=5. (b) 8-OHdG formation, n=5. (c) mRNA levels by qPCR, n=4. (d) EBV viral genomes/cell by qPCR, n=4. *, *P*<0.05, vs CTL group. Results were expressed as mean ± SEM.

**Figure S3. Peptide Tf-D-HKC8 dissociates HKDC1 from VDAC1 in cancer cells, but little effect on normal cells.** Different cells, including HMECS, HANK1 and SW480, were treated by either control (CTL) or Tf-D-HKC8 peptide (0.5µM) for 6 hours, and the cells were used for IP/WB analysis. (a) Representative western blotting bands for IP/WB. (b) Protein quantitation for IP/WB for (a), n=5. *, *P*<0.05, vs CTL group. Results were expressed as mean ± SEM.
