# Supplementary figures and images for: HKDC1 C-terminal based peptides inhibit extranodal natural killer/T-cell lymphoma by modulation of mitochondrial function and EBV suppression
Source: Leukemia. 2020 Mar 23;34(10):2736–48. doi: 10.1038/s41375-020-0801-5 (PMC7515829; doi:10.1038/s41375-020-0801-5)

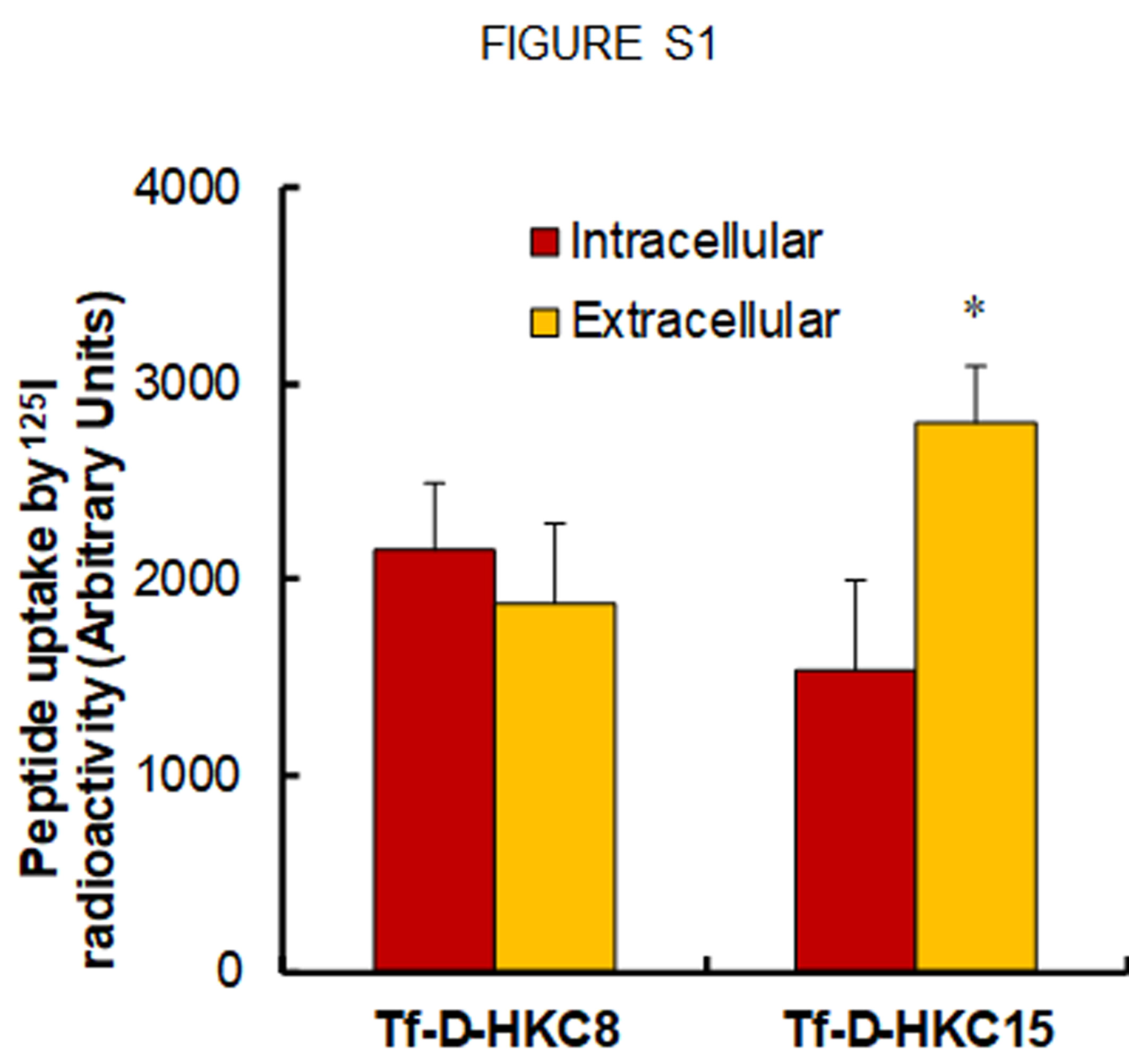

Supplement: Supplementary file 2 — Supplemental Information Figure S1 [file 41375_2020_801_MOESM2_ESM.jpg]

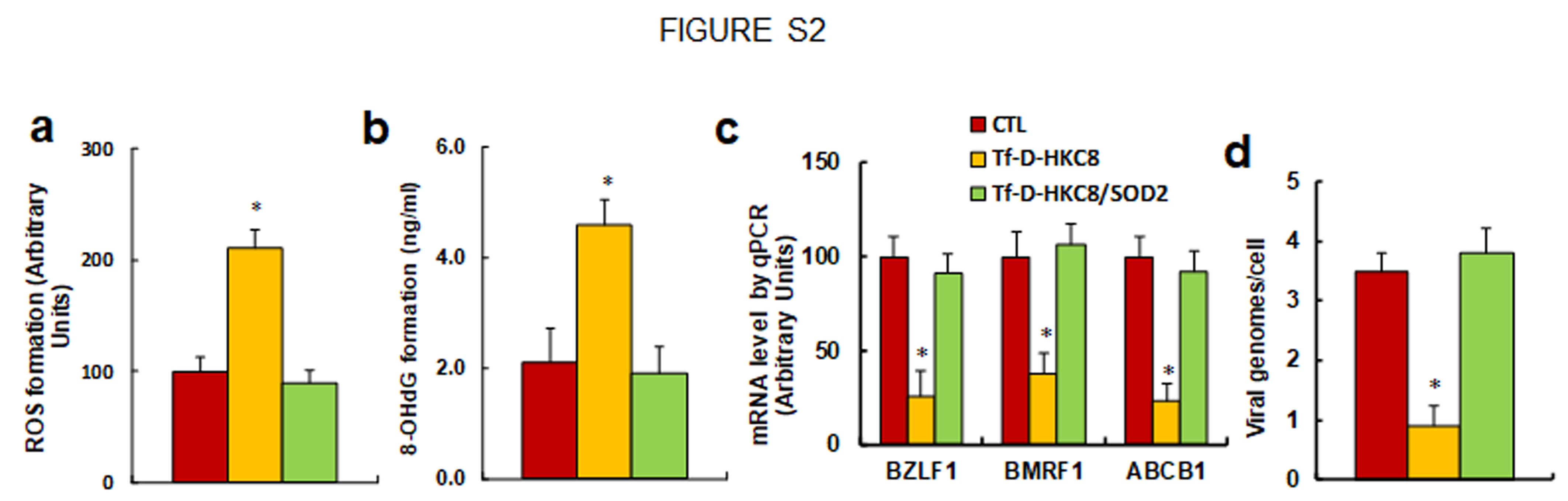

Supplement: Supplementary file 3 — Supplemental Information Figure S2 [file 41375_2020_801_MOESM3_ESM.jpg]

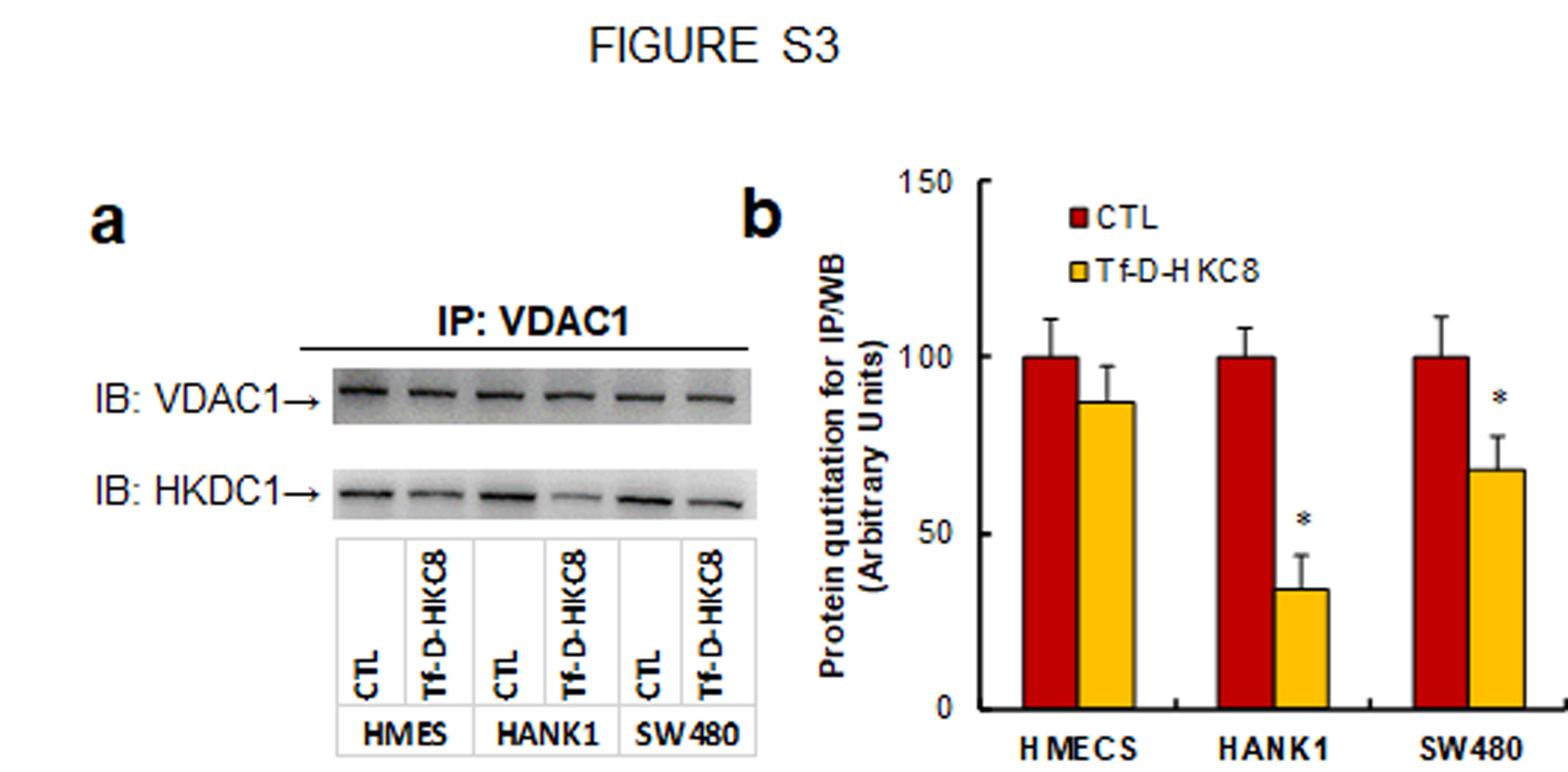

Supplement: Supplementary file 4 — Supplemental Information Figure S3 [file 41375_2020_801_MOESM4_ESM.jpg]
